# Novel Potency Assay for MSC Secretome-Based Treatment of Idiopathic Male Infertility Employed Leydig Cells and Revealed Vascular Endothelial Growth Factor as a Promising Potency Marker

**DOI:** 10.3390/ijms23169414

**Published:** 2022-08-20

**Authors:** Anna Monakova, Georgy Sagaradze, Nataliya Basalova, Vladimir Popov, Vadim Balabanyan, Anastasia Efimenko

**Affiliations:** 1 Faculty of Medicine, Lomonosov Moscow State University, 27/1, Lomonosovskiy Av., Moscow 119192, Russia; 2 Institute for Regenerative Medicine, Medical Research and Education Center, Lomonosov Moscow State University, 27/10, Lomonosovskiy Av., Moscow 119192, Russia

**Keywords:** MSC, secretome, VEGF, Leydig cells, potency assay

## Abstract

Idiopathic male infertility is a highly prevalent diagnosis in developed countries with no specific treatment options. Although empirical medical treatment is widely used to restore male fertility, its efficacy remains limited and inconclusively proven. Therefore, the development of novel therapeutic approaches in this field is a high-priority task. Since the failure of testicular microenvironment components might be involved in the pathogenesis of idiopathic male infertility, application of mesenchymal stromal cells (MSCs) as well as the MSC secretome is worth considering. Previously, we showed that the intratesticular injection of MSCs or the MSC secretome led to the recovery of spermatogenesis at least through replenishing the testicular microenvironment and its maintenance by MSC-secreted paracrine factors. However, the clinical use of such products has been limited to single trials to date. This may be due to the lack of relevant potency tests reflecting mechanisms of action of the MSC secretome in male infertility models. Based on the presumptive MSC secretome mode of action on the testicular microenvironment, we suggest a novel approach to test the potential efficacy of the MSC secretome for idiopathic male infertility treatment. It represents a potency assay based on evaluation of testosterone production by isolated Leydig cells. We demonstrated that the MSC secretome stimulated testosterone secretion by Leydig cells in vitro. We then hypothesized that among the major factors of the MSC secretome, vascular endothelial growth factor (VEGF) could be responsible for the observed effects, which we confirmed by the revealed correlation between the extent of stimulated testosterone production and VEGF concentration in the MSC secretome. The pilot results obtained from the doxorubicin-induced male infertility murine model also indicate the important impact of VEGF in the MSC secretome’s regenerative effects. Utilizing VEGF as a surrogate factor, a novel approach to study the potency of MSC secretome-based products for idiopathic male infertility treatment is suggested. Further validation is required for its implementation into the biopharmaceutical manufacturing process.

## 1. Introduction

Idiopathic male infertility is a highly prevalent diagnosis in developed countries and even worldwide. As its etiology cannot be identified in most cases, common treatment approaches consist of assisted reproductive technology or empirical medical treatment, including hormonal therapy or antioxidants [1]. Indeed, some meta-analyses claim that antioxidant supplements, selective estrogen receptor modulators, and hormones can improve sperm parameters such as the concentration, motility, and morphology, or even increase pregnancy rates [2,3]. However, recent randomized clinical trials contradict these conclusions. In particular, it was shown that antioxidants failed to improve semen parameters, as well as in vivo pregnancy or live birth rates [4]. These and other studies indicate that the problem of effective treatment of idiopathic male infertility has not yet been resolved, so the development of novel approaches is highly demanded.

Since accumulating evidence points to the critical contribution of the testicular cellular components to the pathogenesis of idiopathic male infertility, it is reasonable to pay attention to the therapies that lead to the replenishment and/or maintenance of the testicular microenvironment [5]. Hence, mesenchymal stromal cells (MSCs) represent a promising therapeutic tool. The use of MSCs or MSC-derived products is justified by their ability to protect or reactivate the spermatogonial stem cell (SSC) niche [6,7]. In particular, some experimental data indicate that the application of MSCs led to the recovery of spermatogenesis, at least through influencing SSC microenvironment components such as Leydig cells or Sertoli cells [8,9]. However, only single trials of MSCs for male infertility have been announced to date [6]. This may be due to natural and regulatory aspects of MSC application as a therapeutic tool. In particular, it is necessary to maintain the viability and functionality of cells after injection for their effective use [10]. This is worthy of attention as the risks of unwanted differentiation or tumor formation by the injected MSCs might be an issue for immune-privileged zones [11]. Moreover, the lack of clear characteristic criteria for MSCs and the need to establish relevant disease-specific potency assays restrain clinical translation of MSC-based products [12,13].

The above-mentioned issues can be overcome by switching to the use of the MSC secretome. First, the regenerative effects of MSCs are largely mediated by secreted growth factors, cytokines, extracellular matrix components, biomolecules within extracellular vesicles, and transferred organelles [14,15]. Second, the use of the MSC secretome is devoid of the risks associated with the injection of cells. Moreover, the biological activity of the secretome is easier to maintain during manufacturing, sterilization, and storage [16].

Similar to MSCs, standardization of the secretome potency is not a trivial task, since potency assays should reflect the mechanism of action of the MSC secretome within the testicular microenvironment. However, fractionation of secretome components, as well as the removal of individual factors, facilitates the establishment of the main mechanisms of action using in vitro and in vivo models. Consequently, the quantitative analysis of selected surrogate factors can potentially replace biological potency assays and facilitate the clinical translation of the MSC secretome [17,18,19,20]. In this paper, we propose vascular endothelial growth factor (VEGF) secreted by MSCs as a surrogate marker of MSC secretome potency for restoring male fertility. We based our hypothesis on the results of the pilot in vivo study in which VEGF inhibition abolished the ability of the MSC secretome to promote SSC niche regeneration. We also refer to our own and previously published in vitro studies indicating the activity of the secretome of MSCs and VEGF, in particular in relation to the secretory activity of Leydig cells [21]. The proposed approach is aimed at simplifying the MSC secretome standardization procedure. Further data accumulation and validation are required for adoption.

## 2. Results

### 2.1. MSC Secretome Stimulates Testosterone Secretion by Leydig Cells in a Dose-Dependent Manner

**Figure 1:**
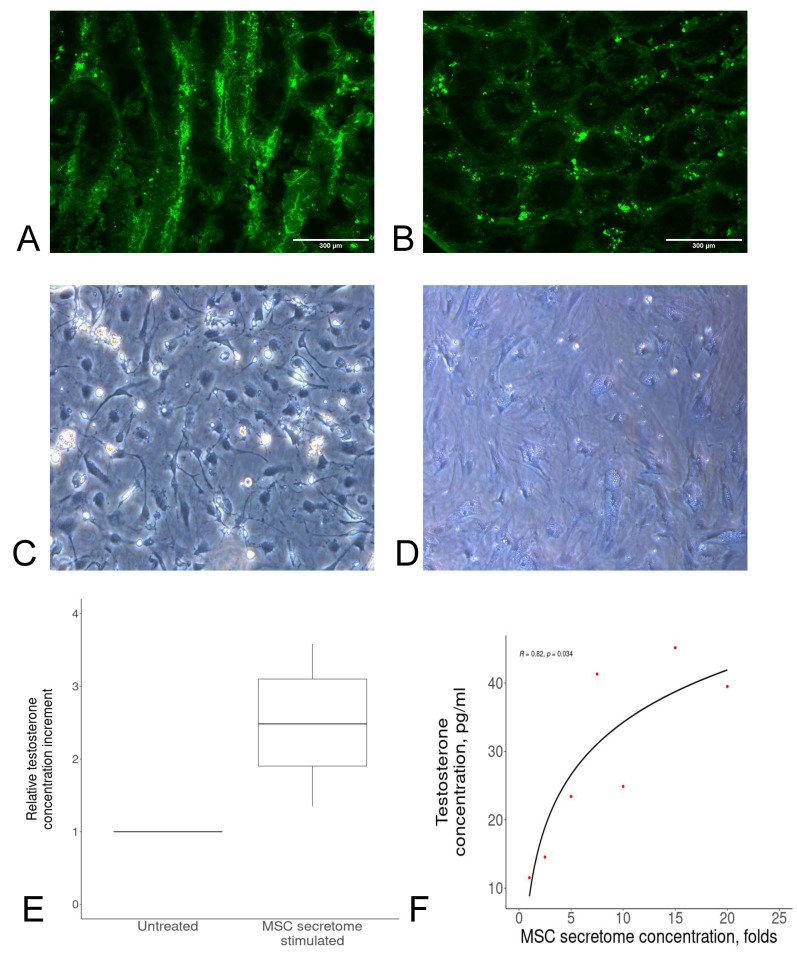
(**A**,**B**) The locally injected mock product was predominantly distributed outside seminiferous tubules: (**A**) collagen (control); (**B**) collagen + GFP. (**C**,**D**) Morphology of Leydig cells: (**C**) 24 h after isolation; (**D**) 96 h after isolation. (**E**) MSC secretome-stimulated Leydig cells secreted more testosterone compared to untreated cells (the value of their potency was equal to one). Data are presented as the median, 25th and 75th percentiles, and minimum and maximum values. (**F**) Testosterone secretion by Leydig cells was directly associated with the MSC secretome concentration; the correlation was calculated using Spearman’s test (R = 0.82, *p* = 0.034). Red dots indicate independent samples.

To facilitate the standardization of MSC secretome-based products for male infertility, relevant potency assays reflecting their mechanism of action are required. Therefore, firstly, we identified the possible cellular targets of the MSC secretome within the testis and SSC niche by injecting a mock product with GFP and found that it was distributed predominantly outside the seminiferous tubules (Figure 1A,B). Considering our previous analysis of the secretome’s putative targets, we suggested that Leydig cells or Sertoli cells can be the main targets of the MSC secretome. In particular, it has been shown that MSC secretome injection led to an increase in the number of Sertoli cells, as well as to increased testosterone release by Leydig cells [22]. Taken together, we considered the latter effect attractive for further potency assay development, as it can be measured by objective instrumental methods such as ELISA.

To test this hypothesis, we isolated Leydig cells following previously published protocols [23] and characterized them (Figure 1C,D). Then, we developed the Leydig cell-based potency assay. In brief, Leydig cells were seeded in DMEM:F12 with 2% FBS and supplements. Two days after Leydig cell isolation, the medium was changed to either DMEM:F12 with supplements (control) or the MSC secretome. Two days after the addition of the MSC secretome, testosterone concentrations were measured in secretome samples of Leydig cells treated with the control medium or the MSC secretome. Based on the results of the proposed assay, it can be assumed that MSC secretome-stimulated Leydig cells secreted more testosterone compared to control cells (*p* < 0.01, compared to the control, the relative potency of the untreated cells was equal to one, Figure 1E). Importantly, the observed effects were dependent on an MSC secretome concentration rate that indicates the suitability of the model to select an optimal dose of the studied product (*p* = 0.034, Figure 1F). We also confirmed the possibility to use Leydig cells after freezing for this assay.

### 2.2. Testosterone Secretion by Leydig Cells Is Directly Related to VEGF Content in MSC Secretome

**Figure 2:**
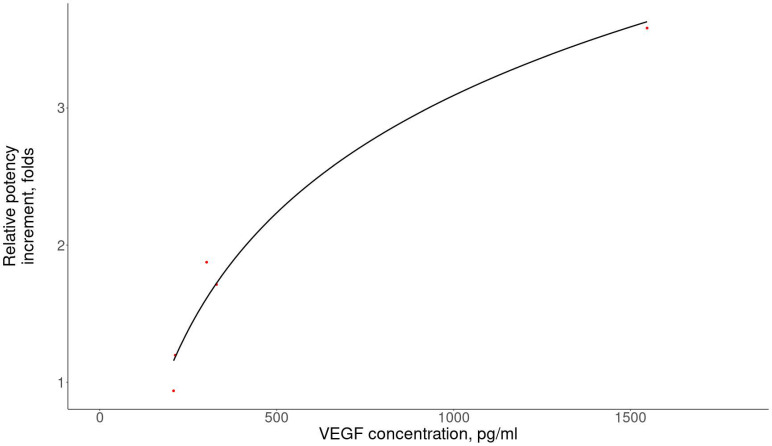
The VEGF concentration in the MSC secretome was directly associated with the potency increment (testosterone secretion) compared to cells from the control group (R = 0.9, *p* = 0.083, Spearman’s test). The Y axis represents the ratio of the testosterone concentration after MSC secretome treatment to the testosterone concentration in the control without the MSC secretome. The correlation was calculated using Spearman’s test. Red dots indicate independent samples.

The next step was to identify the molecules potentially involved in the restoration of spermatogenesis in the proposed MSC secretome potency assay. In particular, VEGF was one of the candidates, since VEGF stimulated testosterone secretion, as shown in the Leydig cell line hypoxic model [21]. According to our data, VEGF could stimulate testosterone secretion by primary Leydig cells in the suggested potency assay in a dose-dependent manner. Notably, a direct significant relationship between the concentration of VEGF and the intensity of testosterone secretion by Leydig cells was confirmed (*p* = 0.083, Figure 2).

### 2.3. Removal of VEGF Abolishes Effects of MSC Secretome Injection in a Murine Model of Doxorubicin-Induced Male Infertility

**Figure 3:**
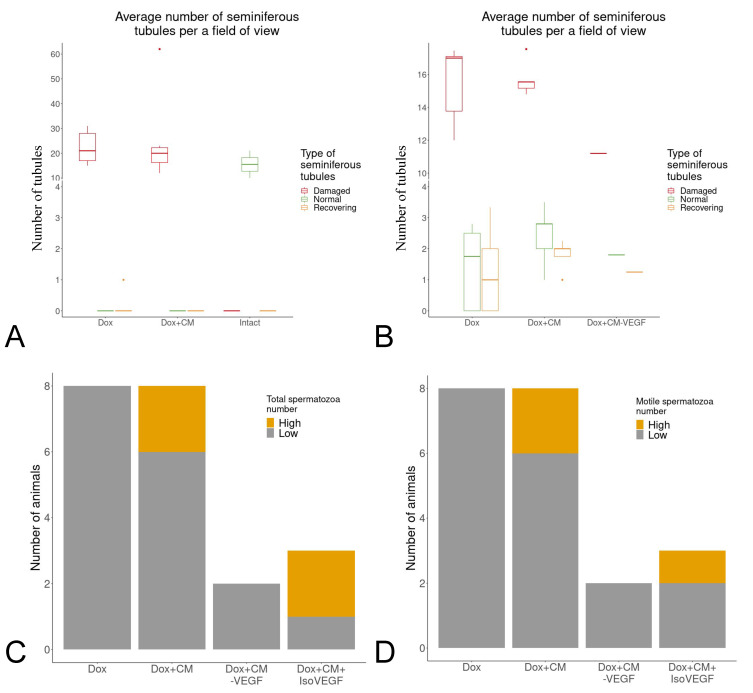
(**A**,**B**) The injection of doxorubicin damaged the seminiferous tubules. A single injection of the MSC secretome did not restore the morphology of the seminiferous tubules 35 days after completion of doxorubicin injections (**A**) but slightly increased the number of regenerating tubules within 150 days after doxorubicin injections (**B**). The median, 25th and 75th percentiles, and minimum and maximum values for each group are shown. (**C**,**D**) Total (**C**) and motile (**D**) spermatozoa fractions measured 150 days after completion of doxorubicin injections. Yellow indicates the number of animals with a total number exceeding the threshold, and gray indicates the number of animals with a total number that does not exceed the threshold. *p* > 0.05 between the Dox + CM group and the Dox + CM-VEGF group on both plots; Fisher’s exact test was used. n = 8 for the Dox + CM group, n = 5 for the Dox + CM group, n = 2 for the Dox + CM-VEGF group, and n = 3 for the Dox + CM + IsoVEGF group.

Despite the contribution of VEGF to the potency of the MSC secretome demonstrated in vitro, it should correspond to the proposed mechanism of action which can only be tested in vivo. To reinforce the results obtained using the in vitro test, we evaluated the role of VEGF in the regenerative effects of the MSC secretome in the doxorubicin-induced male infertility murine model. The injection of doxorubicin led to a significant increase in the number of damaged tubules (Figure 3A) and a decrease in the total and motile fractions of spermatozoa specific for male infertility. Five months after the injection of the MSC secretome, we observed the particular restoration of the motile and total fractions of spermatozoa and a slight increase in the number of regenerating tubules (Figure 3B–D). Notably, inhibition of VEGF with neutralizing antibodies abolished the beneficial effects of the MSC secretome (Figure 3C,D). Taken together, VEGF as a component of the MSC secretome may be one of the key paracrine mediators of testis microenvironment restoration in the model of male infertility. The demonstrated relationship between the amount of VEGF in the composition of the MSC secretome and its potency and efficacy allows us to propose VEGF as a surrogate marker of MSC secretome potency for treating male infertility.

Abbreviations: Intact—animals without any manipulations; Dox—animals with doxorubicin injections; Dox + CM (conditioned medium)—animals with MSC secretome injection after doxorubicin injections; Dox + CM-VEGF—animals with MSC secretome + VEGF antibody injection after doxorubicin injections; Dox + CM + IsoVEGF—animals with MSC secretome + isotype VEGF antibody injection after doxorubicin injections.

## 3. Discussion

Among cases of male infertility, idiopathic male infertility predominates [24]. Empirical therapy with antioxidants, SERMs, or hormones might improve some sperm parameters but does not show a significant effectiveness in pregnancy rates, which serve as the most objective outcome to show the effect of treatment for male infertility [3,4,24,25]. This may be because the pathogenesis of male infertility cannot be fully modified by the aforementioned drugs. For example, proper function of the testicular microenvironment, which is difficult to recover, can be disrupted by such a pivotal pathogenetic factor as oxidative stress. In particular, ROS-associated male infertility may be associated with a disturbance of the follicle-stimulating hormone (FSH)-dependent function of Sertoli cells. Thus, patients with a varicocele associated with elevated ROS levels demonstrated a direct association between post-varicocelectomy levels of inhibin B and improvements in a spermogram [26,27].

However, it is known that Sertoli cells can inhibit the mitogenic activity of Leydig cells in an FSH-dependent manner, and dysregulation of this process in testicular failure is often represented by Leydig cell hyperplasia [28,29]. One of the factors regulating the latter effect may be VEGF, which is produced by Sertoli cells and stimulates testosterone secretion by Leydig cells [21,30]. Despite the ability of Leydig cells to secrete VEGF^33^, this factor can still mediate Sertoli cell-dependent testosterone secretion by Leydig cells [31]. Thus, dysfunctional Sertoli cells would not be able to block excessive Leydig cell proliferation in idiopathic male infertility. At the same time, pharmacological restoration of Sertoli cell function may not be effective, as the number of Leydig cells remains excessive, competing for stimulatory factors, which are thus not able to function properly.

Hence, a reboot of the intercellular interactions between the components of the testicular microenvironment by mimicking a sufficient number of stimuli may help to recover spermatogenesis. This can be achieved by the injection of MSCs, or regulatory factors presented in their secretome, as MSCs are shown to produce many factors important for spermatogenesis maintenance. In particular, MSCs secrete glial-derived growth factor (GDNF), fibroblast growth factor 2 (FGF2), VEGF, and pigment epithelium-derived growth factor (PEDF), which are involved in maintaining the structure or function of testicular microenvironment components [8,32,33]. Moreover, indirect evidence indicates some similarity of Sertoli cells as well as their secretome functions to MSC secretome effects [34,35].

Indeed, both MSCs and components of the MSC secretome demonstrate some beneficial effects in multiple male infertility animal models. However, in order to translate MSC secretome-based products into clinical practice, they should meet predefined quantitative criteria of identity and potency before the products are successfully released for clinical testing or use. In particular, defining the potency remains the main challenge for such products, as potency assays are recommended to represent mechanisms of action by drug regulatory agencies [17]. The complexity of the mechanism of action of such products makes it difficult to determine which product attributes are the most relevant to measuring potency. Therefore, the identification of individual factors associated with the mechanism of action of the MSC secretome may be appropriate. Importantly, if there are significant correlations between instrumentally measured surrogate metrics and the biological activity of the drug, instrumental metrics can be used to measure potency [36]. They can be applied for standardization, since the use of instrumental methods compared to cellular methods will give greater accuracy and be less laborious.

In this study, we unveiled that in the potency assay we developed, testosterone secretion by Leydig cells was directly related to the VEGF content in the MSC secretome. These results correspond to the literature data on the effects of VEGF on Leydig cells, although the involvement of other factors in this process could also be considered [28,37]. Moreover, in the pilot study in vivo, we confirmed that the addition of anti-VEGF antibodies to the injected MSC secretome abolished its beneficial effects on spermatogenesis restoration.

However, we should pay attention to several limitations of our study. Firstly, the experiments performed did not allow us to check whether VEGF alone is sufficient to provide the observed effects of MSC secretome administration: other components of the MSC secretome can also influence its potency [38]. The ability of a single local injection of the MSC secretome to eliminate the proposed pathogenic causes of male infertility, namely, the disproportion of Leydig cells and Sertoli cells, should be further confirmed in more detailed histological analyses. Secondly, in this proof-of-concept study, we did not pay attention to the source of MSCs, age and comorbidities of donors, or methods of cell expansion, although they can influence the potency of the final product in some instances [8,39,40,41,42,43,44,45]. Finally, the results were obtained using MSCs and Leydig cells isolated from different species, which may affect the conclusions. However, we have previously shown in vivo that the injection of the human MSC secretome was sufficient to recover spermatogenesis and the production of functional germ cells in rats with injured spermatogenesis [8]. Importantly, isolation of Leydig cells from rat testicles is more ethically acceptable and yields more material from a single animal, and cells are suitable for use after freezing, improving the reproducibility of the suggested potency assay.

Taken together, based on the application of the suggested Leydig cell-based potency assay, VEGF is proposed as a surrogate marker of MSC secretome potency for the treatment of male infertility. This assay must be validated and further analyzed to be used as a correlative measure of the effectiveness of the MSC secretome for its implementation into the biopharmaceutical manufacturing process.

## 4. Materials and Methods

### 4.1. Isolation of MSCs

Human adipose-derived MSCs were obtained from the biobank of the Institute for Regenerative Medicine, Lomonosov MSU, collection ID: MSU_MSC_AD (https://human.depo.msu.ru, accessed on 1 August 2022). The material was obtained from 8 healthy donors aged 18–53 years. Patients with the presence of autoimmune pathologies, malignant neoplasms (including oncological diseases in their medical history), acute or chronic inflammatory diseases, diabetes, acute or chronic viral and bacterial infections, long-term hormonal or antibiotic therapy, pregnancy, and polyvalent allergies were excluded from this study. All procedures performed with tissue samples from patients were in accordance with the Declaration of Helsinki and approved by the Ethics Committee of Lomonosov Moscow State University (IRB00010587), protocol #4 (2018). MSCs were cultured in 100 mm culture dishes in a medium supporting the growth of undifferentiated mesenchymal progenitor cells (AdvanceSTEM™ Cell Basal Medium, HyClone, USA), containing 10% growth factor supplement (AdvanceSTEM™ Cell Growth Supplement, HyClone, USA) and 100 U/mL of penicillin/streptomycin (Gibco, USA). The medium was changed every 3–4 days. The immunophenotype and differentiation potential of MSCs were analyzed in accordance with previously published criteria [46].

### 4.2. Manufacturing of MSC Secretome

MSCs at passages 4–5 that reached 80% confluency were thoroughly washed 3 times using 10 mL of HBSS without Ca^2+^ and Mg^2+^ and replenished with DMEM without phenol red, with low glucose, 1× Glutamax-I (Gibco, Thermo Fisher Scientific, Carlsbad, CA, USA), and 1× pyruvate solution (Gibco, Thermo Fisher Scientific, Carlsbad, CA, USA). Cells were conditioned in standard culture conditions (5% CO_2_; 37 °C) for 7 days. Then, MSC secretome samples were collected and centrifuged at 300× *g* for 10 min at 4 °C to remove cell debris. The MSC secretome was concentrated 50-fold using a centrifugal ultrafilter with a 10 kDa molecular weight cutoff (VS2062, Sartorius) and then frozen in aliquots at −80 °C for subsequent experiments.

### 4.3. Isolation of Leydig Cells

Eight-month-old male Wistar rats were anesthetized in a desiccator using CO_2_ and a flow rate that displaced at least 20% of the chamber volume per minute. We waited until breathing stopped and there was an absence of reaction upon squeezing a foot pad and then euthanized the rat by cervical dislocation. Testes from the rat were removed using Hanks solution (PanEko, Russia) with 5% antibiotic penicillin/streptomycin (Gibco, Thermo Fisher Scientific, Carlsbad, CA, USA), transferred into 100 mm culture dishes, decapsulated, and then washed with 5 mL of HBSS (PanEko, Russia) 3 times. Then, testes were transferred to 7–10 mL of Dulbecco’s Modified Eagle Medium (Gibco, Thermo Fisher Scientific, Carlsbad, CA, USA) with 2.5 mg/mL trypsin and 10 ug/mL DNAse for 4–10 min at 34–35 °C with shaking. As a result, interstitial cells were dissociated from the seminiferous tubules. To inactivate the enzymes, 15–20 mL of DMEM (Gibco, Thermo Fisher Scientific, Carlsbad, CA, USA) + 10% fetal bovine serum (Gibco, Thermo Fisher Scientific, Carlsbad, CA, USA) was added, mixed gently by inverting the tube, and then left for 5–20 min to allow the tubules to settle down. The liquid supernatant fraction with interstitial cells was moved into a new tube. The procedure of mixing and supernatant removal was repeated 5 times. The obtained cell suspension was centrifuged at 300× *g* and resuspended in DMEM:F12, supplemented with 1× penicillin/streptomycin (Gibco, Thermo Fisher Scientific, Carlsbad, CA, USA), 1× Glutamax-I (Gibco, Thermo Fisher Scientific, Carlsbad, CA, USA), 1× pyruvate (Gibco, Thermo Fisher Scientific, Carlsbad, CA, USA), 2% fetal bovine serum (Gibco, Thermo Fisher Scientific, Carlsbad, CA, USA), and 1× Insulin-Transferrin-Selenium (ITS, PanEko, Russia). Then, the Leydig cell suspension was passed through a 100 µm filter. Leydig cells were seeded in 48-well plates and placed into a CO_2_ incubator at 35 °C.

### 4.4. Modeling the Potency Assay using Leydig Cells

Leydig cells were seeded in 48-well plates in DMEM:F12, supplemented with 1× penicillin/streptomycin (Gibco, Thermo Fisher Scientific, Carlsbad, CA, USA), 1× Glutamax-I (Gibco, Thermo Fisher Scientific, Carlsbad, CA, USA), 1× pyruvate (Gibco, Thermo Fisher Scientific, Carlsbad, CA, USA), 2% fetal bovine serum (Gibco, Thermo Fisher Scientific, Carlsbad, CA, USA), and 1× Insulin-Transferrin-Selenium (ITS, PanEko, Russia). The volume of the medium was 350 µL per well. Then, plates with Leydig cells were placed into the CO_2_ incubator at 35 °C. The next day, all wells were washed three times using 500 µL of HBSS (PanEko, Russia), and the medium was replaced with a fresh version. The next day, the wells were washed three times using 500 µL of HBSS (PanEko, Russia), and the medium was changed to either the MSC secretome or DMEM:F12 without phenol red with a low glucose concentration containing 1× penicillin/streptomycin, 1× Glutamax-I, and 1× pyruvate. Two days later, the Leydig cell secretome was collected and centrifuged at 300× *g* for 10 min. The supernatant was stored at −80 °C.

### 4.5. Enzyme-Linked Immunosorbent Assay (ELISA)

The concentration of testosterone in the samples of the Leydig cell secretome was analyzed using ELISA (CAN-TE-250, Diagnostics Biochem, Canada). The VEGF concentration in the MSC secretome was measured using ELISA for human VEGF (DVE00, R&D Systems).

### 4.6. Doxorubicin-Induced Male Infertility Murine Model

#### 4.6.1. Chemicals

Doxorubicin is a red-orange powder (chemical formula: C27H29NO11, molecular weight: 543.5 g/mol), purchased from the Blokhin National Medical Center of Oncology (Moscow, Russia). For injection in animals, doxorubicin was diluted in 0.9% NaCl.

#### 4.6.2. Animals

The work was performed on mature C57bl/6 male mice with standard weight characteristics. Animal housing and research procedures were conducted in compliance with Directive 201/63/EU and in accordance with the local ethics committee (#90-G). Thirty-six male mice were divided into six groups. Animals were euthanized 35 or 150 days after doxorubicin injection completion. The Intact group consisted of 5 intact mice (2 and 3 for each time period). The Dox group (n = 5 for each time period) received 1 mg/kg of doxorubicin i.p. once in two days for twenty days to reach a cumulative dose of 10 mg/kg. The Dox + CM group (n = 8 for each time period) was treated the same as the Dox group and was also subtunically injected with the MSC secretome using an insulin syringe. The Dox + CM-VEGF group (n = 2, euthanized 150 days after doxorubicin injection completion) was treated the same as the Dox group and was also subtunically injected with the MSC secretome and 0.1 ug/mL of anti-VEGF antibody (ab36424, Abcam, Waltham, MA, USA) using an insulin syringe. The animals from the Dox + CM + isoVEGF group (n = 3, euthanized 150 days after doxorubicin therapy completion) were subtunically injected with the MSC secretome and an isotype control antibody (910801, Biolegend, San Diego, CA, USA) in a 1:10,000 volume concentration using an insulin syringe. The MSC secretome in a 50× concentration was mixed with 2% porcine collagen gel (MakMedi, Moscow, Russia) in the volume ratio of 1:4 to reach a 10× concentration for injection.

### 4.7. Histological Analysis

Testicles were excised together 35 or 150 days after doxorubicin injection completion, placed in buffered 10% neutral formalin solution for 24 h, and then embedded in paraffin. Transverse 1 μm-thick sections of the testicles were cut and placed on Polysine Adhesion Slides (Menzel, Rüthen, Germany). Sections were stained with hematoxylin and eosin (Dako, Carpinteria, CA, USA). Images were captured using a Leica DMi8 with a Leica DFC7000T camera (Leica Microsystems GmbH, Mannheim, Germany) and processed using FIJI software (GitHub Inc., Microsoft, Redmond, WA, USA).

### 4.8. Morphometric Analysis of the Contents of the Epididymis

Epididymes isolated 35 or 150 days after doxorubicin therapy completion were mechanically stirred with scissors in a 5% sterile glucose solution. The resulting suspension was analyzed using Goryaev’s chamber. The numbers of total and moving spermatozoa were calculated.

### 4.9. Phase-Contrast Microscopy

Phase-contrast microscopy was used to observe cells and take photos of randomly selected areas of the cultured cells. Images were captured using a Leica DMIL LED with a Leica MC170 HD camera (Leica Microsystems GmbH, Mannheim, Germany).

### 4.10. Biodistribution Assay

Mice with doxorubicin damage of spermatogenesis (for design, see above) were injected with 50 uL 2% porcine collagen gel (MakMedi, Russia) or with a 50 uL combination of 2% porcine collagen gel (MakMedi, Russia) and the recombinant green fluorescent protein (GFP) in a concentration of 0.1 mg/mL (kindly provided by Sergey Larin and Alexey Kibardin, Laboratory of Molecular Immunology, Dmitry Rogachev National Medical Center of Pediatric Hematology, Oncology and Immunology, Moscow, Russia). One hour later, the animals were euthanized. The testicles were excised, cut in half, and placed in Tissue-Tek freezing liquid. After that, the testicles were frozen in liquid nitrogen and cut on a cryotome to obtain 1 μm-thick histological sections. Images were captured using a Leica DMi8 with a Leica DFC7000T camera (Leica Microsystems GmbH, Mannheim, Germany) and processed using FIJI software [47].

### 4.11. Statistical Analysis

The type of the distribution was analyzed using the Shapiro–Wilk test. Comparisons between two groups were conducted using the Mann–Whitney U test. Statistical significance between multiple groups was analyzed using ANOVA for parametric data or the Kruskal–Wallis test for non-parametric data. Post hoc analysis was not applied since no statistically significant differences were found using ANOVA or Kruskal–Wallis tests. Fisher’s exact test was conducted to analyze qualitative data. Differences were considered significant when * *p* < 0.05.

## Data Availability

Data are available on request.
